# An Immunocompetent Mouse Model for MLL/AF9 Leukemia Reveals the Potential of Spontaneous Cytotoxic T-Cell Response to an Antigen Expressed in Leukemia Cells

**DOI:** 10.1371/journal.pone.0144594

**Published:** 2015-12-11

**Authors:** Kana Hasegawa, Satomi Tanaka, Fumihiro Fujiki, Soyoko Morimoto, Hiroko Nakajima, Naoya Tatsumi, Jun Nakata, Satoshi Takashima, Sumiyuki Nishida, Akihiro Tsuboi, Yoshihiro Oka, Yusuke Oji, Atsushi Kumanogoh, Haruo Sugiyama, Naoki Hosen

**Affiliations:** 1 Department of Cancer Immunology, Osaka University Graduate School of Medicine, Suita, Osaka, Japan; 2 Department of Functional Diagnostic Science, Osaka University Graduate School of Medicine, Suita, Osaka, Japan; 3 Department of Cancer Immunotherapy, Osaka University Graduate School of Medicine, Suita, Osaka, Japan; 4 Department of Respiratory Medicine, Allergy and Rheumatic Disease, Osaka University Graduate School of Medicine, Suita, Osaka, Japan; 5 Department of Immunopathology, WP1 Immunology Frontier Research Center, Osaka University, Suita, Osaka, Japan; 6 Department of Cancer Stem Cell Biology, Osaka University Graduate School of Medicine, Suita, Osaka, Japan; B.C. Cancer Agency, CANADA

## Abstract

Leukemia differs substantially with respect to stromal milieu from tumors that progress locally as solid masses, and the physiological importance of immunosurveillance in leukemia remains unclear. However, currently available mouse leukemia models have critical limitations in the context of analyzing immunological regulation of leukemia development. In this study, we transferred mouse MLL/AF9 leukemia-initiating cells into immunocompetent recipient mice without any pre-conditioning such as irradiation, and then analyzed the spontaneous T cell response to an immunogenic antigen expressed in leukemia cells. When the minimum numbers of leukemia-initiating cells for engraftment were transferred, leukemia cells were eradicated by the adaptive immune response in most, if not all, wild-type mice, but not in *Rag2* ^-/-^ recipient mice, which lack adaptive immunity. By contrast, mice transplanted with larger numbers of leukemia cells always developed leukemia. In mice with advanced leukemia, antigen-specific CTLs were also expanded, but were unresponsive to antigen stimulation and expressed high levels of PD-1 and LAG-3. These results provide the first clear demonstration that the spontaneous CTL response to a tumor-cell antigen has the potential to eradicate leukemia, whereas antigen-specific CTLs are exhausted in animals with advanced leukemia. This immunocompetent mouse leukemia model provides a useful platform for developing effective immunotherapies against leukemia.

## Introduction

The spontaneous cytotoxic T lymphocyte (CTL) response to tumor-cell antigens suppresses tumor development in solid tumor models [1–4]. However, it remains unclear whether CTL-mediated immune surveillance can inhibit systemic hematological malignancies, which differ substantially with respect to stromal milieu from tumors that progress locally as solid masses. Local production of “danger signals” from apoptotic tumor cells in the solid tumor microenvironment is important for induction of an adaptive T-cell response against tumor cell–associated antigens[5–8]. In leukemia, however, local inflammation is not likely to be induced. Previous reports showed that T cells become tolerant to antigens expressed in intravenously injected leukemia cells, whereas subcutaneous inoculation of leukemia cells efficiently induced a CTL response to these antigens [9, 10]. In addition, in a mouse leukemia model generated by transplant of Bcr/Abl- and NUP98/HOXA9-transduced hematopoietic progenitor cells into sub-lethally irradiated recipients, CTLs recognizing antigens expressed in leukemia cells were exhausted [11].

In human leukemia patients, CTLs specific for leukemia-associated antigens (LAAs) are detected in peripheral blood, e.g., by staining with MHC-peptide tetramers, suggesting that the spontaneous CTL response to LAAs may counteract leukemia progression [12–15]. LAA-specific CTL lines established from healthy donors can certainly kill leukemia cells *in vitro* [16–21]. In addition, CTL lines recognizing Tax, a virus-derived exogenous antigen, can target virus-induced human leukemia cells *in vivo* in a xenograft model [22]. However, the significance of other types of human LAA-specific CTLs *in vivo* remains unclear.

These findings raise the question of whether the spontaneous CTL response to antigens expressed in leukemia cells can suppress leukemia progression even if immunogenic antigens are expressed in leukemia cells. However, currently available mouse leukemia models have critical limitations in the context of analyzing the immunological regulation of leukemia development. Leukemia models generated by transplantation of oncogene-transduced hematopoietic progenitor cells are convenient, and therefore frequently used. In these models, however, irradiation of the recipients suppresses the immune response and also induces tissue damage, resulting in non-physiological inflammation. Transgenic or knock-in mouse models that spontaneously develop leukemia are also widely used; however, in these models, antigen expression throughout the target organs (including normal tissues) is likely to change the endogenous T cell response to tumors [23–25]. Furthermore, in most transgenic models, additional oncogenic events are needed for full transformation, and consequently the courses of leukemia development are variable [26].

MLL/AF9, a fusion gene generated by the t(9;11) translocation [27] that is responsible for a subset of human acute monocytic leukemia, can transform hematopoietic progenitor cells (HPCs)[28]. MLL/AF9-transduced HPCs are for the most part distinct from leukemia cell lines, in that they possess the potential not only to initiate leukemia but also to differentiate into mature progeny *in vivo* [29, 30]. Because the MLL/AF9 oncogene confers self-renewal potential on HPCs, MLL/AF9-expressing HPCs (MLL/AF9-HPCs) can expand without limit [29, 31], thus enabling us to transfer clonal leukemia-initiating cells into large numbers of recipient mice.

Neo-antigens with high immunogenicity are generated as a result of genetic mutations in cancer [32]. In mouse solid tumor models, highly immunogenic antigens appear as a result of genetic mutations and induce the CTL response strongly enough to eradicate tumors [4]. In patients responding to tumor-infiltrating lymphocyte (TIL) transfer [33] or check-point antibody therapy, CTLs recognize neo-antigens derived from genetic mutations [34]. Neo-antigens may be derived from passenger mutations, and are therefore likely to differ from patient to patient. In this study, in order to compare the CTL response to a single antigen expressed on leukemia cells between different animals, we used ovalbumin (OVA) as a model antigen. OVA is convenient as a model antigen because CTLs recognizing OVA can be easily identified using the MHC-OVA peptide tetramer. In addition, CTLs recognizing OVA can be obtained from OT-1 transgenic mice [35], which express an OVA-specific T cell receptor in T cells and are used for functional analysis.

In this study, we established MLL/AF9 leukemia-initiating cells that express OVA as a model tumor antigen and have the potential to engraft in bone marrow (BM) of recipient mice without any pre-conditioning. By transferring MLL/AF9-OVA leukemia-initiating cells into non-irradiated immunocompetent mice, we investigated whether the spontaneous antigen-specific CTL response could suppress development of leukemia, and also how leukemia develops despite the presence of a CTL response to an immunogenic leukemia antigen.

## Materials and Methods

### Mice

C57BL/6 mice (from 6- to 8- week old, female) were purchased from CREA Japan (Osaka, Japan). Rag2^-/-^ mice were kindly provided by Dr. Mamoru Ito (Central Institute for Experimental Animals, Kawasaki, Japan). OT-1 transgenic mice were obtained from the center of animal resources in Kumamoto University. All animal experiments in this study were approved by the administrative panel on laboratory animal care in Osaka University.

### Retroviral transduction to BM progenitor cells and transplantation

MLL-AF9 cDNA[27] and OVA cDNA[36], which were kindly gifted from Cleary ML (Stanford University) and Bevan MJ (University of Washington), were subcloned into MSCV-Neo vector and MSCV-IRES-GFP vector, respectively. Retroviral stocks were produced by transient transfection of retroviral vectors to the Plat-E packaging cell line[37] (a kind gift from Kitamura T, Tokyo University) using Lipofectamine 2000 (Invitrogen, Carlsbad, CA, USA). C-kit^+^ BM cells were purified from 4- to 8-week-old mice using anti-c-kit microbeads (Miltenyi Biotec, Bergisch Gladbach, Germany), cultured overnight in RPMI 1640 medium supplemented with 10% fetal calf serum, 10 ng/ml SCF, 10 ng/ml IL-3, and 10 ng/ml IL-6 (Pepro Tech, Rocky Hill, NJ, USA), and then infected with MLL/AF9-Neo retroviral supernatants in the presence of 4 μg/ml polybrene for 24 hours. Two days after the infection, cells were plated in methylcellulose medium (Stem Cell Technologies, Vancouver, Canada) containing 10 ng/ml SCF, 10 ng/ml IL-6, 10 ng/ml GM-CSF, 10 ng/ml IL-3, and 400μg/ml G418 (Roche, Basel, Switzerland). After 5 days of culture, colonies were pooled, and then 10^4^ cells were replated in the same medium. At the end of the third round culture, a colony was plucked up from methylcellulose and transferred to liquid culture in the media containing 10 ng/ml SCF, 10 ng/ml IL-3, and 10 ng/ml IL-6. The resultant MLL/AF9 leukemia cells were infected with either MSCV-OVA-ires-EGFP or MSCV-EGFP virus, and then EGFP^+^ cells were FACS-sorted using FACS Aria II (BD Biosciences, San Jose, CA, USA). Establishment of mouse MLL/AF9 leukemia cells was approved by the institutional committee for recombinant DNA experiments of Osaka University. Immortalized hematopoietic progenitor cells expressing MLL/AF9 (and OVA) were expanded *in vitro*, and transplanted into recipient mice by retro orbital injection. For minimizing mice suffering and distress, we used inhaled anesthesia (isoflurane) when leukemia cells were injected into mice. Health status of the mice transplanted with leukemia cells were carefully examined twice a week. When clinical signs of illness (hunchback or reduced motility) were observed or more than 50% of PB cells were GFP^+^ leukemia cells, mice were diagnosed as leukemia and sacrificed by excess anesthesia with pentobarbital. If leukemia became not apparent 3 months after leukemia cell transfer, mice were sacrificed and subjected to the analysis.

### ^51^Cr releasing assay

Splenocytes from the OT-1 transgenic mice were cultured in the media containing 10% FBS, 45% RPMI1640 medium, 45% AIM-V, 65IU/ml IL2, and 7.5 μg/ml SIINFEKL peptide for 5 days. Then, CD8^+^ T cells purified using CD8 T cell enrichment kit (BD Biosciences) were used as effector cells. Target cells were labeled with Chromium-51 (Cr^51^) by incubating for 90 minutes, and then co-cultured with the OVA-specific CD8^+^ T cells for 4 hours. An aliquot of supernatant was removed, and the amount of radioactivity was measured with a gamma counter. Percent of specific lysis was calculated as follows: [(experimental release–spontaneous release) / (maximum release–spontaneous release)] × 100.

### Flow cytometry

Cells were treated with CD16/32 antibody (93: BioLegend, San Diego, CA, USA) to block non-specific mAb binding, and then stained with the following fluorochrome-conjugated antibodies: CD8α (53–6.7: BioLegend), α-PD-1 (29F.1A12: BioLegend), α-MHC I (H-2K^b^) (AF6-88.5.5.3: eBioscience, San Diego, CA, USA), α-OVA_257-264_ (SIINFEKL) peptide bound to H-2K^b^ (eBio25-D1.16: eBioscience), CD117 (c-kit) (2B8: BioLegend), CD11b (M1/70: BioLegend), CD45.1 (A20: BioLegend), and CD45.2 (104: BioLegend). OVA-specific CTLs were stained with PE-conjugated H-2K^b^ OVA Tetramer-SIINFEKL (MBL, Nagoya, Japan) and Alexa647-CD8 (KT15: MBL) according to the manufacturer’s instructions. Cells were analyzed on FACS Aria II or FACS Canto II (BD Biosciences).

For the analysis of cytokine production, cells were cultured in the medium containing 10% FBS, 45% RPMI1640 medium, 45% AIM-V medium, and 5 μg/ml of Brefeldin A (Sigma-Aldrich, St. Louis, MO, USA) with or without 7.5 μg/ml of SIINFEKL peptide for 4 hours at 37°C. Cells were then fixed and stained for intracellular cytokines using the Cytofix/Cytoperm kit (BD Biosciences) and antibodies against IFN-γ (XMG1.2: BD Biosciences) and TNF-α (MP6-XT22: BioLegend).

### Statistical analysis

Statistical analysis in each experiment was performed by two-sample t-test. Differences with P < 0.05 were considered significant.

## Results

### Establishment of MLL/AF9 leukemia cells that express an immunogenic antigen and have the potential to engraft to non-irradiated mice

**Fig 1:**
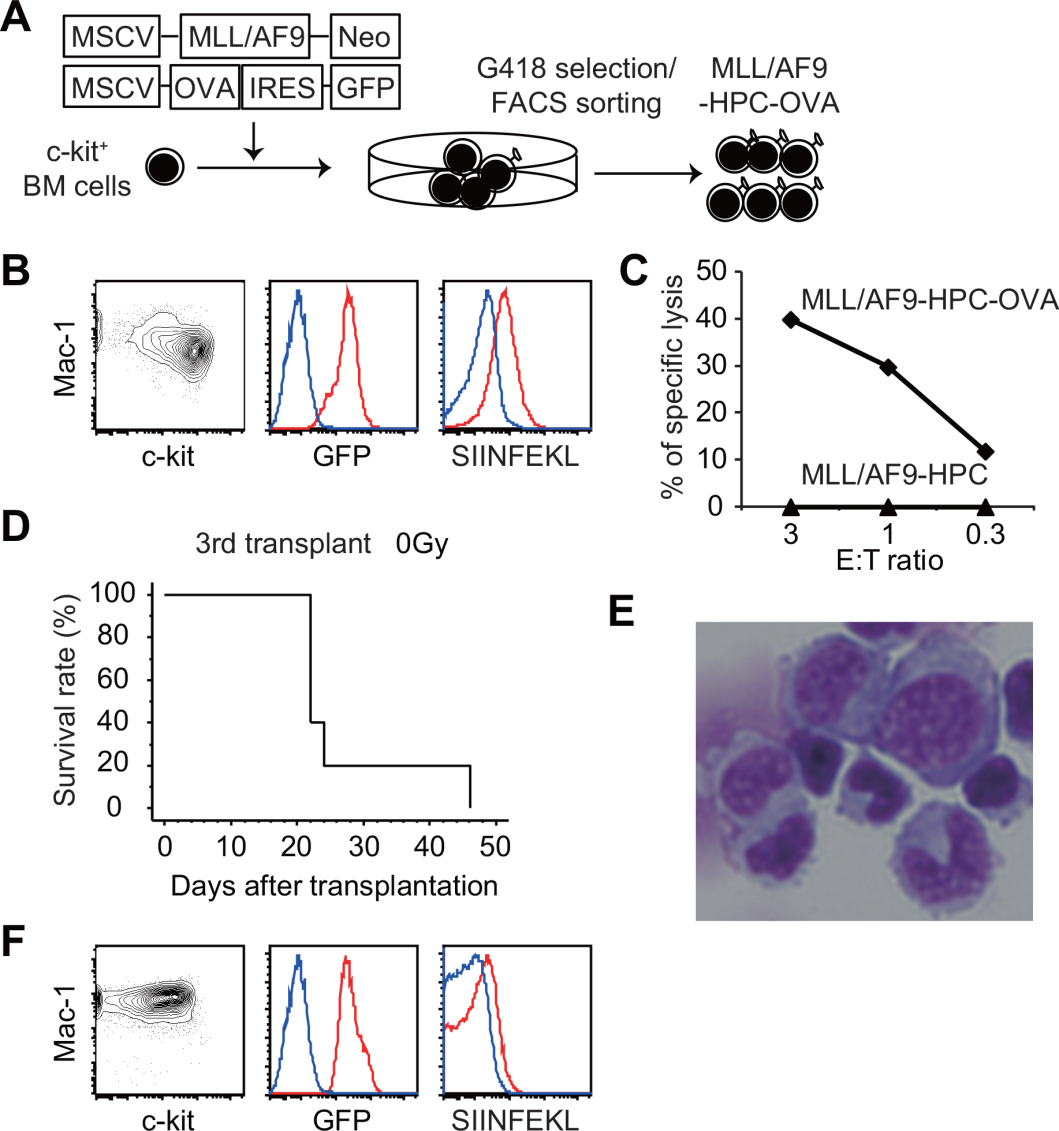
Establishment of MLL/AF9 leukemia cells expressing a model tumor antigen. (A) Scheme for establishment of MLL/AF9-HPC-OVA cells. (B) FACS analysis of MLL/AF9-HPC-OVA cells. Blue lines represent MLL/AF9-HPC cells. (C) ^51^Cr-release assay using activated CD8^+^ T cells from the OT-1 mouse as effector cells. MLL/AF9-HPC-OVA or MLL/AF9-HPC cells were used as targets. E:T ratio denotes effector-per-target ratio. (D) Kaplan–Meier curves for overall survival of wild-type mice that received 1 × 10^6^ MLL/AF9-HPC-OVA cells in the third transplant (n = 5). Recipient mice were not irradiated. Results from the first and second transplants are shown in S1 Fig. (E) May–Giemsa staining of MLL/AF9-OVA leukemia cells that developed in non-irradiated recipients (Magnification: 400×). (F) Flow-cytometry analysis of GFP^+^ BM cells from mice with leukemia. Blue lines represent MLL/AF9-HPC cells.

MLL/AF9-immortalized hematopoietic progenitor cells (HPCs) expressing OVA as a model tumor antigen (MLL/AF9-HPC-OVA cells) were established as previously reported [38, 39], with some modifications. MSCV-MLL/AF9-Neo and MSCV-OVA-IRES-GFP were retrovirally transduced into c-kit^+^ mouse HPCs. Cells with both neomycin resistance and GFP expression were selected and expanded in the presence of SCF, IL3, and IL6, and named MLL/AF9-HPC-OVA cells (Fig 1A). MLL/AF9-HPC-OVA cells were Mac1^+^, and separated into c-kit–positive and–negative fractions as previously reported [30] (Fig 1B). All MLL/AF9-HPC-OVA cells expressed OVA-IRES-GFP and presented OVA-derived SIINFEKL peptide on H-2K^b^ (Fig 1B). In addition, MLL/AF9-HPC-OVA cells were lysed *in vitro* by activated OVA-specific CD8^+^ T cells derived from OT-1 transgenic mice (Fig 1C).

Lethally (9 Gy) irradiated wild-type recipients transplanted with 10^6^ MLL/AF9-HPC-OVA cells developed leukemia, whereas non-irradiated recipients did not (Figure A in S1 Fig). Leukemia cells that developed in the lethally irradiated recipients could be secondarily transferred to sub-lethally (4.5 Gy) irradiated recipients (Figure B in S1 Fig). Next, we transferred 10^6^ leukemic BM cells from the secondary recipients into non-irradiated recipients, and found that all the recipient mice developed myeloid leukemia (Fig 1D–1F). We then transferred 10^6^ GFP^+^ leukemia cells from the third recipients into multiple non-irradiated recipients. Leukemia cells obtained from BM of these recipients were collected, named MLL/AF9-OVA leukemia cells, and used for further experiments.

### Spontaneous regression of leukemia was observed in the presence, but not in the absence, of adaptive immunity

**Fig 2:**
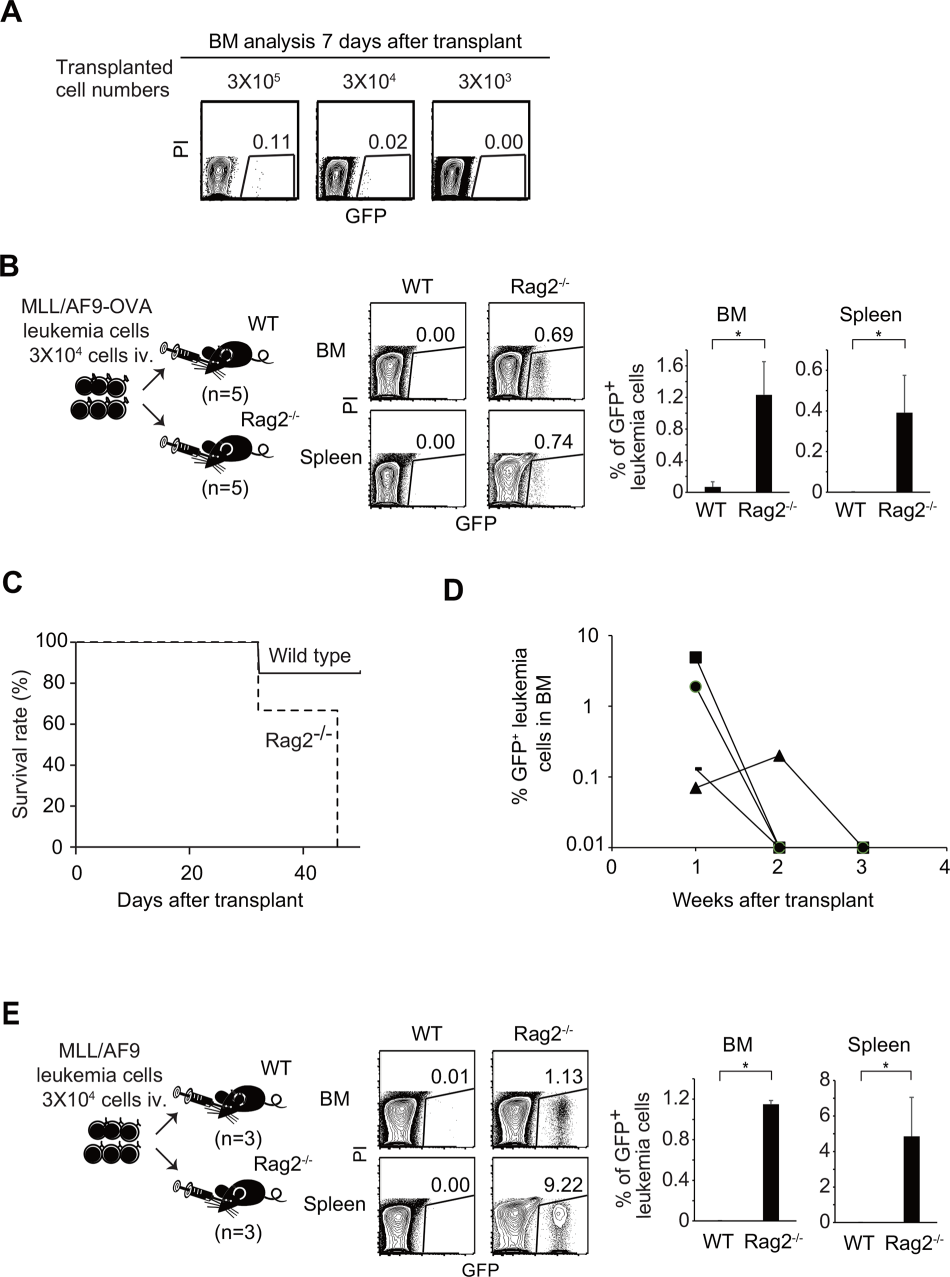
Spontaneous regression of leukemia was observed in the presence, but not in the absence, of adaptive immunity. (A) Flow-cytometry analyses of BM cells of non-irradiated wild-type recipients 7 days after transplantation with different numbers (3 × 10^3^, 3 × 10^4^, or 3 × 10^5^) of MLL/AF9-OVA leukemia cells. (B) FACS analysis of BM from non-irradiated wild-type or *Rag2* ^-/-^ mice transplanted with 3 × 10^4^ MLL/AF9-OVA leukemia cells. Mice were analyzed 3 weeks after transplant. (C) Kaplan–Meier curves for overall survival of non-irradiated wild-type (n = 7) or Rag2^-/-^ (n = 3) recipients transplanted with 3 × 10^4^ MLL/AF9-OVA leukemia cells. (D) Percentages of GFP^+^ leukemia cells in BM after transplantation into non-irradiated wild-type mice were examined every week. Each dot and line corresponds to a recipient mouse. The results of four mice in which leukemia spontaneously regressed (Exp. 3 in Table 1) are shown. (E) FACS analysis of BM from non-irradiated wild-type or *Rag2* ^*-/-*^ mice transplanted with 3 × 10^4^ of MLL/AF9 leukemia cells (OVA-). Mice were analyzed 3 weeks after transplant.

Next, to analyze the immune response without overwhelming it with too many leukemia cells, we reduced the number of leukemia cells transferred into recipient mice to the minimum dose required for engraftment. To determine the minimum number of MLL/AF9-OVA leukemia cells required for engraftment in non-irradiated hosts, different numbers (3 × 10^3^, 3 × 10^4^, and 3 × 10^5^) of GFP^+^ MLL/AF9-OVA leukemia cells were transplanted into non-irradiated recipients, and BM cells were analyzed 1 week after transplant. In mice transplanted with 3 × 10^3^ leukemia cells, no engraftment was detected. By contrast, tiny but distinct GFP^+^ cell populations were detected in the BM of mice transplanted with 3 × 10^4^ leukemia cells (Fig 2A).

**Table 1:** Transplant experiments performed in this study.

Exp	Cell	Cell numbers	Recipients	Numbers of recipients	Leukemia development
1	MLL-AF9/OVA leukemia	3 × 10^4^	Wild-type	5	1/5*
	MLL-AF9/OVA leukemia	3 × 10^4^	*Rag2* ^*-/-*^	5	5/5*
2	MLL-AF9/OVA leukemia	3 × 10^4^	Wild-type	7	1/7
	MLL-AF9/OVA leukemia	3 × 10^4^	*Rag2* ^*-/-*^	3	3/3
3	MLL-AF9/OVA leukemia	3 × 10^4^	Wild-type	7	3/7
4	MLL-AF9 leukemia (OVA-)	3 × 10^4^	Wild-type	3	0/3*
	MLL-AF9 leukemia (OVA-)	3 × 10^4^	*Rag2* ^*-/-*^	3	3/3*
5	MLL-AF9/OVA leukemia	3 × 10^4^	Wild-type	9	1/9
6	MLL-AF9/OVA leukemia	3 × 10^6^	Wild-type	3	3/3
7	MLL-AF9/OVA leukemia	3 × 10^6^	Wild-type	3	3/3

*: Mice were sacrificed 3 weeks after transfer of leukemia cells. In these cases, mice were diagnosed as having leukemia when expansion of a distinct GFP^+^ leukemia cell population was detected in BM.

To determine whether the CTL response eradicated leukemia cells that had engrafted *in vivo*, we transplanted 3 × 10^4^ MLL/AF9-OVA leukemia cells into either wild-type recipients (n = 5) or *Rag2* ^-/-^ recipients (n = 5), which lack adaptive immunity (Fig 2B, Exp. 1 in Table 1). When BM cells were analyzed 3 weeks after transfer, the percentages of GFP^+^ leukemia cells in BM were 0.07 ± 0.07% and 1.22 ± 0.42% in wild-type and *Rag2* ^-/-^ recipients, respectively (p<0.05). In spleen of *Rag2* ^*-/-*^, but not wild-type, recipients, distinct leukemia cell population was detected (0.39 ± 0.18%). The same experiments were performed using additional wild-type recipients (n = 7) or *Rag2* ^-/-^ recipients (n = 3), which were observed until they developed clinically apparent leukemia (Fig 2C, Exp. 2 in Table 1). All *Rag2* ^-/-^ recipients became moribund due to leukemia within 50 days. By contrast, six of the seven wild-type recipients did not develop leukemia. To confirm that MLL/AF9-OVA leukemia cells were engrafted and then eliminated in the wild-type recipients, we transplanted 3 × 10^4^ MLL/AF9-OVA leukemia cells into another set of wild-type recipients (n = 7, Exp. 3 in Table 1), and then aspirated and analyzed BM cells every week. In all mice, GFP^+^ leukemia cells engrafted in BM 1 week after transplant. In four of the seven mice, GFP^+^ leukemia cells diminished and finally disappeared 3 weeks after transplant (Fig 2D). Taken together, these results indicate that the adaptive immune response could eradicate leukemia cells that had engrafted. In a part of the wild-type mice, CTLs lost in the competition with expansion of the leukemia cells and leukemia developed finally. The frequency of leukemia was not constant when mice were transplanted with the minimum numbers of leukemia cells for engraftment (Table 1), probably because these experiments used the threshold numbers of leukemia cells capable of evading immune surveillance and inducing leukemia.

Finally, we investigated whether the leukemia cells were eliminated by adaptive immunity in the absence of OVA expression in leukemia cells. MLL/AF9 leukemia cells lacking OVA expression were established by the same methods used for MLL/AF9-OVA cells. We transplanted 3 × 10^4^ MLL/AF9 leukemia cells into either wild-type recipients (n = 3) or *Rag2* ^-/-^ recipients (n = 3) (Fig 2E, Exp. 4 in Table 1). When the *Rag2* ^*-/-*^ recipients were analyzed 3 weeks after transfer, the percentages of GFP^+^ leukemia cells were 1.15 ± 0.04% and 4.85 ± 2.20% in BM and spleen, respectively. By contrast, no leukemia cells were detected in BM and spleen of the wild-type recipients. In addition, significant increase in the numbers of total or activated (CD69^+^) CD8 T cells were observed in spleen, but not in BM, of the mice transplanted with MLL/AF9 leukemia cells irrespective of OVA expression (S2 Fig). These results indicated that MLL/AF9 leukemia cells were eliminated by the adaptive immune system in the absence of OVA expression.

### Functional CTLs specific for the antigen expressed in leukemia cells were highly expanded in mice that did not develop leukemia

**Fig 3:**
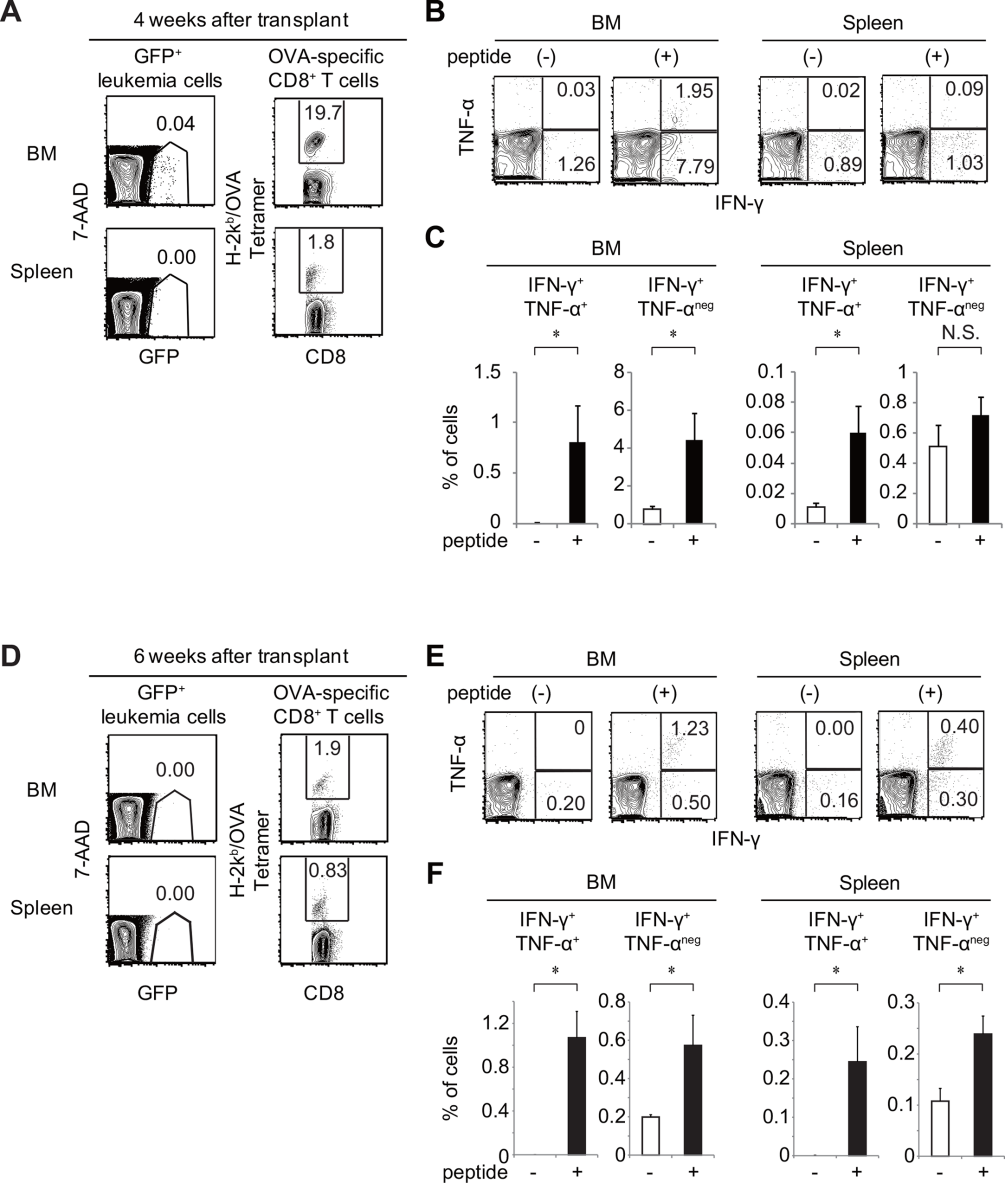
Functional CTLs specific for the antigen expressed in leukemia cells were highly expanded in mice that did not develop leukemia. Analysis of BM and spleen cells from mice 4 weeks (A–C, n = 5) or 6 weeks (D–F, n = 3) after transplant with 3 × 10^4^ MLL/AF9-OVA leukemia cells. (A, D) Flow-cytometry analysis of the frequencies of GFP^+^ leukemia cells in the 7AAD^-^ whole BM or spleen cells and those of H-2K^b^/OVA tetramer-positive cells in CD8^+^ T cells. (B, E) Flow-cytometry analysis of cytokine production by CD8^+^ BM and spleen T cells, with or without SIINFEKL peptide stimulation. (C, F) Percentages of IFN-γ- and/or TNF-α-producing cells in BM or spleen CD8^+^ T cells, with or without SIINFEKL peptide stimulation. *: p < 0.05.

Next, we again transplanted MLL/AF9-OVA leukemia cells (3 × 10^4^ cells/mouse) into another set of non-irradiated recipient mice (n = 9, Exp. 5 in Table 1) to determine whether functional OVA-specific CTLs were expanded in mice in which leukemia had regressed. Four weeks after transplant, six mice were sacrificed for the analysis of BM and spleen cells. Five of the six mice that showed no signs of leukemia (splenomegaly or anemia) were extensively analyzed. In these non-leukemic mice, GFP^+^ leukemia cells were rare, but could be identified by FACS analysis (Fig 3A). The frequencies of H-2K^b^/OVA tetramer-positive cells in CD8^+^ T cells were 9.7 ± 3.1% (1.9–19.7%) in BM and 0.9 ± 0.2% (0.6–1.8%) in spleen (Fig 3A), indicating that OVA-specific CTLs were highly expanded in BM, and to a lesser extent in spleen. To determine whether OVA-specific CTLs expanded in these mice could respond to antigen stimulation, we measured the production of IFN-γ and TNF-α in CD8^+^ T cells after stimulation with SIINFEKL peptide. The frequencies of TNF-α^+^ IFN-γ^+^ cells among CD8^+^ T cells significantly increased (p < 0.05), from 0.01 ± 0.01% to 0.81 ±0.36% in BM and from 0.01±0.00% to 0.06 ± 0.02% in spleen. The frequency of TNF-α^-^ IFN-γ^+^ cells among CD8^+^ T cells also significantly increased from 0.77 ± 0.18% to 4.41 ± 1.41% in BM (Fig 3B and 3C). These results demonstrated that CTLs specific for the antigens expressed in leukemia cells were highly expanded in these mice, and could respond to antigen stimulation.

The remaining recipient mice were analyzed 6 weeks after transplant. In all of these mice, GFP^+^ leukemia cells disappeared completely (Fig 3D). The frequencies of OVA-specific CTLs that stained positively with the H-2K^b^/OVA tetramer were still high in both BM and spleen. The frequencies of H-2K^b^/OVA tetramer-positive cells among CD8^+^ T cells were 1.71± 0.36% in BM and 0.54± 0.15% in spleen (Fig 3D). We also examined production of IFN-γ and TNF-α in CD8^+^ T cells after stimulation with SIINFEKL peptide. The percentages of TNF-α^+^ IFN-γ^+^ cells in CD8^+^ T cells significantly increased (p < 0.05) from 0.00% to 1.07 ± 0.24% in BM and from 0.00% to 0.24 ± 0.09% in spleen (Fig 3E and 3F). The frequencies of TNF-α^-^ IFN-γ^+^ cells in CD8^+^ T cells also increased, from 0.20 ± 0.01% to 0.57 ± 0.16% in BM and from 0.11 ± 0.02% to 0.24 ± 0.04% in spleen (Fig 3E and 3F). These results demonstrated that antigen-specific CTLs persisted after leukemia cells disappeared.

### CTLs specific for the antigen expressed in leukemia cells were also expanded, but could not suppress disease progression in mice with advanced leukemia

**Fig 4:**
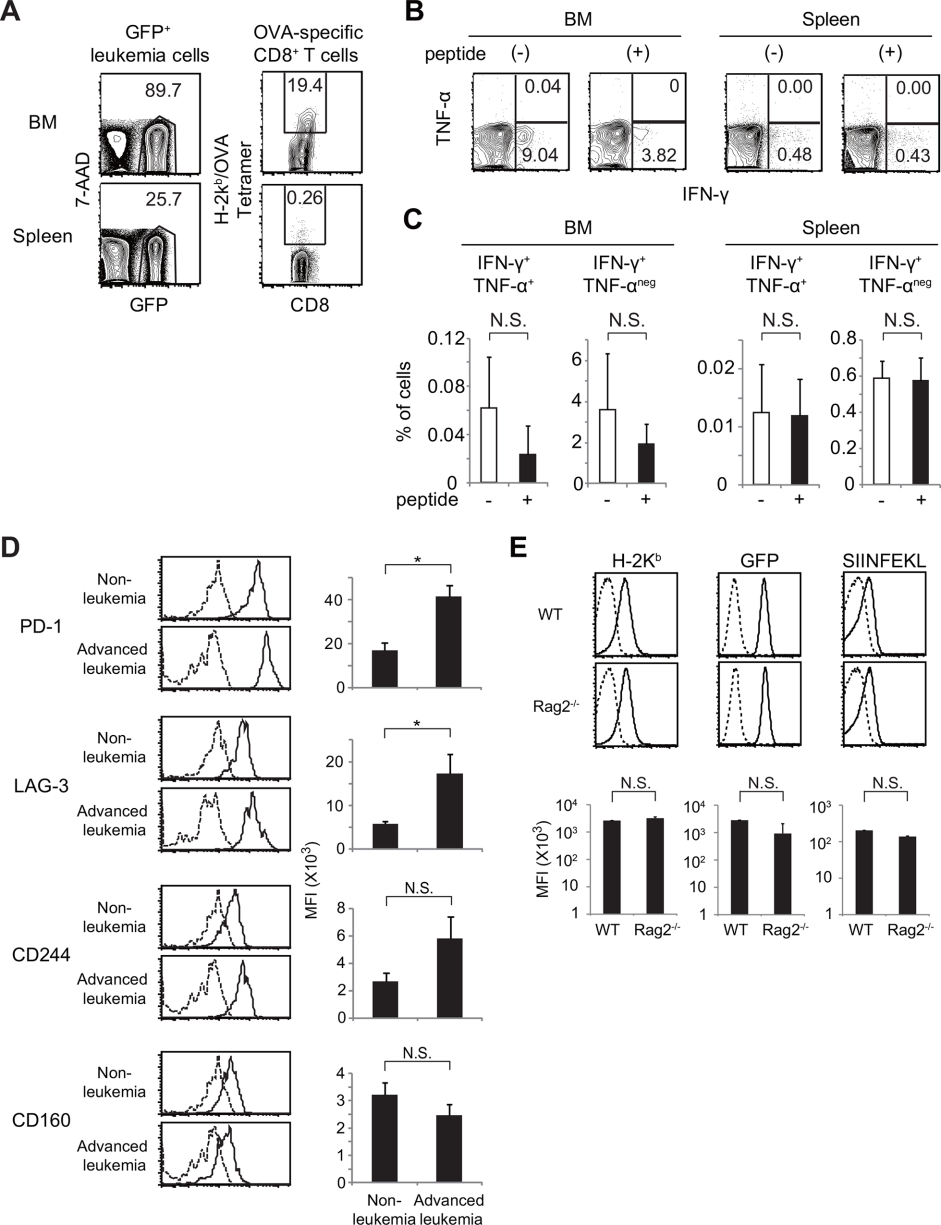
In mice with advanced leukemia, CTLs specific for the antigen expressed in leukemia cells were also expanded, but could not suppress disease progression. (A-C) Analysis of BM and spleen cells from mice with advanced MLL/AF9-OVA leukemia (A) Flow-cytometry analysis of the frequencies of GFP^+^ leukemia cells among the whole BM or spleen cells and the frequencies of H-2K^b^/OVA tetramer-positive cells among CD8^+^ T cells. (B) Flow-cytometry analysis of cytokine production by CD8^+^ T cells in BM and spleen with or without SIINFEKL peptide stimulation. (C) Percentages of IFN-γ- and/or TNF-α-producing cells among CD8^+^ BM or spleen T cells, with or without SIINFEKL peptide stimulation (n = 3). *: p < 0.05 (D) Analysis of the expression of T-cell exhaustion–associated markers in H-2K^b^/OVA tetramer-positive CD8^+^ T cells. BM cells from non-leukemic mice and mice with advanced leukemia were analyzed (n = 3 for each). Representative flow-cytometry analysis and bar graphs for mean fluorescence intensities (MFI) are shown. Dotted lines represent isotype controls.*: p < 0.05, N.S.: not statistically significant. (E) Analysis of the expression of H-2K^b^, GFP, and the presentation of SIINFEKL peptide in leukemia cells that developed in wild-type or *Rag2* ^-/-^ recipients. Representative flow-cytometry analysis and bar graphs for MFI are shown. Dotted lines represent isotype controls.

When large numbers of leukemia cells (3 × 10^6^) were transplanted into non-irradiated mice, all the mice developed leukemia (Exp. 6 and 7 in Table 1). We examined whether antigen-specific CTLs could be detected in mice with advanced leukemia. In the leukemic mice (n = 3; one of them was the leukemia mice in Exp. 5), the frequencies of GFP^+^ leukemia cells were 94.5±2.6% (89.7–98.6%) in BM and 57.4±16.2% (25.7–78.8%) in spleen (Fig 4A). The frequencies of H-2K^b^/OVA tetramer-positive cells among CD8^+^ T cells were 32.7 ±19.3% (8.0–70.8%) in BM and 0.85 ±0.59% (0.26–2.0%) in spleen (Fig 4A), indicating that OVA-specific CTLs had expanded greatly in BM and to a lesser extent in spleen. We also measured the production of IFN-γ and TNF-α in CD8^+^ T cells after stimulation with SIINFEKL peptide in these mice. High frequencies of TNF-α^+^ IFN-γ^+^ cells and TNF-α^-^ IFN-γ^+^ CD8^+^ T cells were observed before *in vitro* antigen stimulation (Fig 4B and 4C), suggesting that the CTLs might respond to antigen expressed by leukemia cells *in vivo*. However, after stimulation with SIINFEKL peptide in vitro, the frequencies of TNF-α^+^ IFN-γ^+^ cells and TNF-α^-^ IFN-γ^+^ cells among CD8^+^ T cells decreased from 0.06 ±0.04% to 0.02 ± 0.02% and from 3.6 ±2.7% to 1.9 ±1.0% in BM (Fig 4B and 4C). In spleen, the frequencies of TNF-α^+^ IFN-γ^+^ cells and TNF-α^-^ IFN-γ^+^ cells did not change upon antigen stimulation (Fig 4B and 4C). These results indicate that many of the antigen-specific CTLs in advanced leukemia mice might have been already stimulated *in vivo* and could not respond further to antigen stimulation *in vitro*.

We next compared the expression of T-cell exhaustion markers[40] on OVA-specific CTLs in BM between mice in which leukemia regressed (Fig 3A, called “non-leukemic mice” hereafter) and those with advanced leukemia (Fig 4A). Expression levels of PD-1 in the OVA tetramer^+^ CD8^+^ T cells were significantly higher in mice with advanced leukemia than in non-leukemic mice (Fig 4D). LAG-3 expression was also significantly higher in CTLs from the mice with advanced leukemia. Expression levels of CD244 and CD160, which are expressed in severely exhausted T cells[40], did not significantly differ between non-leukemic mice and mice with advanced leukemia (Fig 4D).

Finally, we investigated whether antigen loss or reduction was observed in leukemia cells that developed in non-irradiated wild-type recipients. Expression levels of H-2K^b^ and GFP did not significantly differ between the leukemia cells that developed in wild-type recipients and those that developed in *Rag2* ^*-/-*^ recipients (Fig 4E). The amounts of SIINFEKL peptide presented on H-2K^b^ were also similar. These results indicate that the mechanism of immune evasion in this leukemia model did not involve loss or reduction of antigen expression.

## Discussion

In this study, we transferred mouse MLL/AF9 leukemia-initiating cells expressing a model tumor cell antigen into immunocompetent mice without any pre-conditioning such as irradiation. Using this system, we demonstrated that the CTL response to an antigen expressed in leukemia cells has the potential to induce spontaneous leukemia regression, as reported in a human MLL/AF9 leukemia patient [41]. Our result differs from those of previous reports showing that T-cell tolerance was induced when C1498 leukemia cells were intravenously injected [9, 10]. However, a difference in the location where leukemia cells engrafted might be responsible for this discrepancy. C1498 leukemia cells predominantly engrafted in the liver, whereas MLL/AF9 leukemia cells engrafted and expanded mainly in BM, which is the most physiologically relevant location for leukemia. We speculate that BM-residing dendritic cells, which have the potential to induce efficient adaptive immune responses even in the absence of secondary lymphoid organs [42], might take up antigens derived from leukemia cells and induce an adaptive immune response in the MLL/AF9 leukemia model.

The MLL/AF9-OVA model is convenient for studies of immunological regulation of leukemia. MLL/AF9 leukemia-initiating cells can be expanded without limit *in vitro*, enabling easy transfer of clonal leukemia-initiating cells into multiple recipient mice. By contrast, it is difficult to perform similar experiments with most other leukemia-associated fusion genes such as AML1/ETO, because leukemic or pre-leukemic cells expressing these genes cannot be expanded *in vitro*. In addition, by using OVA as a model antigen, CTL response to an antigen expressed in leukemia cells can be easily analyzed both quantitatively and qualitatively. One critical question that remains is whether a highly immunogenic antigen such as OVA is in fact expressed in leukemia cells. While leukemias generally harbor relatively few mutations [43], highly immunogenic neo-antigens may be found in subsets of leukemia or MDS patients, e.g., those with complex karyotypes.

The MLL/AF9-OVA model will be a good platform for testing new immunotherapies for leukemia. Prevention of leukemia development or relapse is important in patients who have pre-leukemic disease (e.g. myelodysplastic syndrome [MDS]) or whose leukemia is in remission after treatment. To date, however, no effective method has been established for achieving such prevention. Our results suggest that immunotherapies that enhance CTL-mediated immune surveillance have the potential to suppress leukemia development at stages when the leukemia burden is minimal. Immune checkpoint antibodies, such as anti-CTLA4 [44] or anti-PD-1 [45], can enhance the CTL response to solid tumors by “loosening the brakes” on the immune response to cancers, which originate from self-tissues. This strategy is very effective, although the effects are not specific to tumor immunity. Thus, we expect that efficient methods for accelerating tumor antigen-specific CTL response will eventually be established. One such strategy would be vaccination against LAAs, an approach that has yielded promising results in some studies [15, 46, 47]. To further develop more effective cancer vaccines targeting LAAs, it will be important to understand which antigen-presenting cells (APCs) take up the antigen and how those APCs induce the antigen-specific CTL response. The MLL/AF9-OVA leukemia model offers an excellent platform for future analyses aimed at answering these questions.

It would be possible to conduct further experiments using the MLL/AF9-OVA leukemia model. For example, it will be interesting to analyze the CTLs that infiltrate myeloid sarcomas, which are sometimes observed in advanced MLL/AF9 leukemia, and compare them with those in BM or spleen. It will be also important to understand the role of co-operating mutations, e.g. in RAS [48] or FLT3 [49], in the presence of the adaptive immune response. These additional mutations may increase the immunogenicity of leukemia cells by yielding immunogenic epitopes or altering the expression of immune-modulatory molecules [50]. In addition, the changes in proliferation or survival of leukemia cells resulting from such mutations are important factors that determine whether immune control is successful or not.

In addition to CTLs, NK cells also play substantial roles in immunosurveillance during leukemia development. We recently reported that MLL-ENL leukemia developed in *Rag2* ^-/-^ *γc* ^-/-^ mice more frequently and rapidly than *Rag2* ^-/-^ mice, suggesting that NK cells can eliminate leukemia cells even in the absence of adaptive immunity [39]. NK cell–mediated elimination of leukemia is likely explain why as many as 3 × 10^4^ leukemia cells need to be transplanted to induce leukemia even in *Rag2* ^-/-^ recipients. Although we did not observe increase of NK cells in BM or spleen 3 weeks after transfer of MLL/AF9 or MLL/AF9-OVA leukemia cells (S2 Fig), NK cells might increase at earlier time point. NK cells likely eliminate leukemia cells in the early phase of leukemia development, whereas CTLs are the major effectors at later phase. It will be important to determine how we can efficiently induce a CTL response to antigens derived from leukemia cells that are destroyed by NK cells. The MLL/AF9-OVA leukemia model may be useful for addressing this issue.

Consistent with previous reports [9–11], we found that antigen-specific CTLs were exhausted in mice with advanced leukemia. In these mice, antigen-specific CTLs were expanded, but could not eradicate leukemia cells. Furthermore, their CTLs expressed PD-1 and LAG-3, but not CD244 and CD160, at significantly higher levels than those of non-leukemic mice, a phenotype resembling exhaustion during chronic virus infection [40]. These results suggest that leukemia cells escaped from the antigen-specific CTL attack by inducing CTL exhaustion. Therefore, prevention or reversion of CTL exhaustion, e.g., by immune checkpoint antibodies, should be considered in immunotherapy against advanced leukemic disease, as previously suggested [9–11].

In summary, we established an immunocompetent mouse model for MLL/AF9 leukemia. Using this model, we showed that a spontaneous CTL response to an antigen expressed in leukemia cells was efficiently induced and could eradicate leukemia cells. We also confirmed that antigen-specific CTLs in mice with advanced leukemia were exhausted, as previously reported. These results suggest that immunotherapies that enhance the CTL response to leukemia antigens have the potential to prevent leukemia development, but should be performed while the leukemia burden is minimal. The immunocompetent mouse leukemia model presented in this study offers a promising platform for development of effective immunotherapies.

## Supporting Information

S1 Fig Establishment of MLL/AF9-OVA leukemia cells with the potential to initiate leukemia in non-irradiated recipients. (A, B) Kaplan–Meier curves for overall survival of wild-type mice that received 1 × 10^6^ MLL/AF9-HPC-OVA cells in the first (A) and second (B) transplants (n = 5 for each group). Recipient mice were lethally irradiated (9 Gy) in the first transplant experiment, and sub-lethally irradiated (4.5 Gy) in the second.
(EPS)

S2 Fig CD8 T cells and NK cells in spleen and BM of mice transplanted with MLL/AF9 leukemia with or without OVA expression. (A) Representative results of FACS analysis of splenocytes from non-irradiated wild-type mice transplanted with MLL/AF9-OVA or MLL/AF9 (OVA-) leukemia cells. Mice transplanted with the same numbers of un-transduced BM mononuclear cells from other mice were also analyzed as controls. Numbers indicate the percentages of the gated cells in the GFP^-^ splenocytes. (B) Percentages of CD8^+^ T cells, CD69^+^CD8^+^ activated T cells, and CD3^-^NK1.1^+^ NK cells in GFP^-^ splenocytes or BM cells are shown as bar graphs.
(EPS)
